# Genomic signatures of selection for resistance to stripe rust in Austrian winter wheat

**DOI:** 10.1007/s00122-021-03882-3

**Published:** 2021-06-14

**Authors:** Laura Morales, Sebastian Michel, Christian Ametz, Hermann Gregor Dallinger, Franziska Löschenberger, Anton Neumayer, Simone Zimmerl, Hermann Buerstmayr

**Affiliations:** 1 Institute of Biotechnology in Plant Production, Department of Agrobiotechnology, University of Natural Resources and Life Sciences Vienna, Tulln, Austria; 2 Saatzucht Donau GmbH & CoKG, Probstdorf, Austria

## Abstract

**Key message:**

We combined quantitative and population genetic methods to identify loci under selection for adult plant resistance to stripe rust in an Austrian winter wheat breeding population from 2008 to 2018.

**Abstract:**

Resistance to stripe rust, a foliar disease caused by the fungus *P. striiformis* f. sp. *tritici*, in wheat (*Triticum aestivum* L.) is both qualitatively and quantitatively controlled. Resistance genes confer complete, race-specific resistance but are easily overcome by evolving pathogen populations, while quantitative resistance is controlled by many small- to medium-effect loci that provide incomplete yet more durable protection. Data on resistance loci can be applied in marker-assisted selection and genomic prediction frameworks. We employed genome-wide association to detect loci associated with stripe rust and selection testing to identify regions of the genome that underwent selection for stripe rust resistance in an Austrian winter wheat breeding program from 2008 to 2018. Genome-wide association mapping identified 150 resistance loci, 62 of which showed significant evidence of selection over time. The breeding population also demonstrated selection for resistance at the genome-wide level.

**Supplementary Information:**

The online version contains supplementary material available at 10.1007/s00122-021-03882-3.

## Introduction

Stripe rust is an economically important foliar disease of wheat (*Triticum aestivum* L.) caused by the fungus *P. striiformis* f. sp. *tritici* (*Pst*). Breeding resistant varieties are the most effective strategy for mitigating yield losses due to stripe rust (Chen 2020). Qualitative resistance to stripe rust in wheat is controlled by both qualitative resistance genes (R-genes) and quantitative trait loci (QTL) with small to moderate effects. More than 100 QTL have been associated with seedling resistance, adult plant resistance, and high temperature adult plant resistance in dozens of mapping populations and diversity panels (Rosewarne et al. 2008; Zegeye et al. 2014; Ye et al. 2019), and more than 80 *Yr* R-genes have been mapped or proposed to date (Waqar et al. 2018; Blake et al. 2019). Although *Yr* genes can provide complete or nearly complete protection against specific *Pst* races, they can easily break down with genetic changes in *Pst* populations (Poland et al. 2009; Chen 2020). For example, the emergence of the *Warrior* pathotype, which has overcome several widely deployed *Yr* genes, has caused devastating losses across Europe in the past decade (Buerstmayr et al. 2014; Hovmøller et al. 2016; Klymiuk et al. 2020; Tehseen et al. 2020). In contrast, small- and moderate-effect QTL provide partial, race non-specific resistance that tends to be more durable over time (Poland et al. 2009; Chen 2020). Information on *Yr* genes and QTL associated with stripe rust can be used for marker-assisted selection (Chen 2020) and to enhance genomic selection models (Juliana et al. 2017; Muleta et al. 2017).

Here, we analyzed historical stripe rust and genotyping data from an active Austrian winter wheat breeding program across 2008–2018. We employed genome-wide association (GWA) mapping to identify QTL associated with adult plant resistance to stripe rust within and across years and assessed their dynamics in allele frequencies and effects over the 11-year period to test for selection at the locus and genome-wide levels.

## Materials and methods

### Phenotypic and genotypic data

Here, we analyzed a historical stripe rust dataset from the winter wheat breeding program of Saatzucht Donau GmbH & CoKG (Probstdorf, Austria). In total, 20,529 genotypes (12,844 recombinant inbred lines, 1638 doubled haploids, and 6047 advanced lines and registered varieties) were evaluated in 71 trials in 53 locations from 2008 to 2018 (Table 1). Because the plant material was part of an active breeding program, most genotypes were only evaluated in one plot in one trial (Table 1). To account for within-trial spatial variation, a check plot design was used, in which at least one genotype was replicated within each trial (Kempton 1984).

**Table 1:** Total number of plots, trials, and genotypes with/without sequencing data within and across years from 2008 to 2018

Year	Plots	Trials	All genotypes				Sequenced genotypes			
			Total	RIL	DH	Other	Total	RIL	DH	Other
2008	1103	1	962	670	12	280	47	18	0	29
2009	2065	1	1177	674	18	485	93	50	0	43
2010	2325	1	1672	1113	12	547	182	130	0	52
2011	2915	1	1789	1095	122	572	243	162	0	81
2012	3072	1	1701	1048	107	546	288	200	0	88
2013	3421	3	1468	1060	17	391	266	172	10	84
2014	12,363	24	3353	2024	733	596	1458	881	474	103
2015	11,485	17	4134	2295	243	1596	1298	933	163	202
2016	11,188	14	3848	2438	163	1247	1387	1032	54	294
2017	4037	6	1465	382	51	1032	638	348	16	274
2018	5425	2	3507	2173	516	818	1639	862	485	292
Across	59,399	71	20,529	12,844	1638	6047	5233	3481	1004	748

*RIL* recombinant inbred line, *DH* doubled haploid, *Other* advanced lines and registered varieties

Each year, the Institute for Plant Protection in Field Crops and Grassland (Julius Kühn Institute, Kleinmachnow, Germany) provided urediniospores from a mixture of *Pst* pathotypes. The inoculum was then propagated on seedlings of susceptible genotypes in the greenhouse at the Saatzucht Donau research station in Reichersberg, Austria. One trial per year was grown in the Reichersberg disease nursery (location ID = LOC01), where plots were spray-inoculated with urediniospore suspension at the EC29 and EC30 growth stages (Leivermann and Brockerhoff 2015) (Online Resource 1). All other trials relied on natural infection. Each plot was scored for adult stripe rust resistance on a 1 (most resistant) to 9 (most susceptible) scale at 1–3 timepoints after symptoms became apparent on susceptible lines (Online Resource 1).

Genotypes (minimum F5 stage) with good agronomic performance (e.g., lodging resistance, yield, spike morphology), grain quality, and disease resistance (e.g., powdery mildew, Septoria nodorum blotch, stripe rust) were pre-selected for DNA sequencing. From the pre-selected material, a final subset of 5233 genotypes representing the diversity of the breeding program was chosen for sequencing and downstream genomic analysis (Table 1). Leaf samples from a minimum of ten plants per genotype were collected during early summer, and DNA was extracted as described by Saghai-Maroof et al. (1984). The DNA samples were genotyped with a custom 6 K Illumina marker array (Illumina, Inc., San Diego, CA, the USA) and with DArTseq (Diversity Arrays Technology Pty Ltd, Canberra, Australia) genotyping-by-sequencing (GBS) technology (Akbari et al. 2006; Elshire et al. 2011) and single nucleotide polymorphisms (SNPs) were then called using proprietary software. SNP genotypes were coded in terms of alternate alleles “a” and “A”, where − 1 = aa (homozygous “a” allele), 0 = Aa (heterozygous), and 1 = AA (homozygous “A” allele). Missing SNP data was imputed with the “missForest” package (Stekhoven and Bühlmann 2012) in R (R Core Team 2020). To estimate imputation accuracy, 5% of the non-missing SNP data was masked (set to missing) and the dataset was imputed again, resulting in 94.4 ± 3.0% of correctly imputed masked SNPs. After filtering for minor allele frequency > 5% and call rate > 90%, a final set of 9744 SNPs was available for downstream genomic analyses (Online Resource 2). To generate a physical map, we used the nucleotide BLAST tool on the Wheat@URGI portal (Alaux et al. 2018) to compare the marker sequences against the IWGSC RefSeq v2.0 assembly (Appels et al. 2018). The physical position of each SNP was determined by the BLAST query with the greatest coverage value.

### Phenotypic analysis

To adjust for spatial variation in each stripe rust score (stripe rust was scored at 1–3 different timepoints) within each trial, we fit a general linear model with genotype as a random effect and row and column effects modeled as two-dimensional P-splines and then estimated heritability using the “SpATS” package (Rodríguez-Álvarez et al. 2018) in R (R Core Team 2020). For the score with the greatest heritability in each trial, we fit a generalized linear model with genotype as a fixed effect and row and column effects modeled as two-dimensional P-splines with the “SpATS” package (Rodríguez-Álvarez et al. 2018) in R (R Core Team 2020) and then extracted the spatially adjusted stripe rust values (plot-level fitted values) for further analysis (Online Resource 1). We used the “lme4” package (Bates et al. 2015) in R (R Core Team 2020) to fit within-year (2013–2018) and across-year (2008–2018) mixed models with spatially adjusted stripe rust values as the response and genotype and trial as random effects.

We extracted the variance components from each model and estimated broad-sense heritability (*H*^2^) as $H^{2} = \sigma_{{\text{G}}}^{2} /\left( {\sigma_{{\text{G}}}^{2} + \sigma_{\varepsilon }^{2} /p_{h} } \right)$, where *σ*^2^_G_ is the genotypic variance, *σ*^2^_*ε*_ is the error variance, and *p*_*h*_ is defined as $p_{h} = n/\sum\nolimits_{i = 1}^{n} {(1/p_{i} )}$, where *n* is the number of genotypes, and *p*_*i*_ is the number of plots for the *i*th genotype (Holland et al. 2003). We also extracted the genotype best linear unbiased predictors (BLUPs) to estimate phenotypic correlations between years.

### Genome-wise association

Because of the unbalanced nature of the dataset, we used methods that maximize statistical power for GWA in unbalanced studies (George and Cavanagh 2015; Xue et al. 2017; Chen et al. 2021). For within-year GWA, we used the one-stage method, in which a mixed model is fit with plot-level phenotypes as the response and environmental (e.g., trial, year, location), genotypic (e.g., line, family), genetic background (e.g., relationship/kinship matrices, population structure components), and SNP information as fixed or random effects (Xue et al. 2017; Chen et al. 2021). Plant breeding experiments can include large numbers of individuals and/or trials, making one-stage GWA computationally intensive when complex variance–covariance structures (e.g., relationship/kinship matrices) are included to control for background genetic effects (George and Cavanagh 2015; Xue et al. 2017). As such, the more common approach for GWA in plant systems has been the two-stage approach, in which (1) the plot phenotypes are regressed against environmental and genotypic terms and (2) the predicted genotypic means are then used as the phenotype in the GWA model including SNP and genetic background effects (George and Cavanagh 2015; Xue et al. 2017). Two-stage analysis can result in biased estimates in unbalanced studies, but methods have been developed to improve effect estimation when one-stage analysis is not computationally feasible (Möhring and Piepho 2009; Piepho et al. 2012; George and Cavanagh 2015; Xue et al. 2017). For across-year GWA, we employed a weighted two-stage analysis, which has been shown to closely approximate the results of one-stage analysis (Möhring and Piepho 2009; George and Cavanagh 2015; Xue et al. 2017).

For one-stage within-year (2013–2018) GWA, we fit mixed models with spatially adjusted stripe rust values as the response, SNP as a fixed effect, and genotype (only genotypes with SNP data) and trial as random effects using the “sommer” package (Covarrubias-Pazaran 2016) in R (R Core Team 2020). For within-year GWA from 2008 to 2012, the trial term was not included, as stripe rust was only evaluated in one inoculated trial in each of these years.

For two-stage across-year GWA, we first fit a mixed model with spatially adjusted stripe rust values as the response, genotype (all genotypes) as a fixed effect, and trial as a random effect using the “breedR” package (Muñoz and Sanchez 2020) in R (R Core Team 2020). We extracted the genotype best linear unbiased estimates (BLUEs) and standard errors (SE) of the genotype BLUEs from the model and then calculated the variances (*σ*^*2*^) of the genotype BLUEs as $\sigma^{2} = \left( {{\text{SE}}\sqrt n } \right)^{2}$, where *n* is the number of observations per genotype (Online Resource 3). In the second stage, we used the “sommer” package (Covarrubias-Pazaran 2016) in R (R Core Team 2020) to fit a GWA mixed model with genotype BLUEs as the response, SNP as a fixed effect, and genotype (only genotypes with SNP data) as a random effect.

For both within-year and across-year GWA, the variance of the genotype term was modeled as ***K****σ*^2^_a_, where ***K*** is the realized additive relationship matrix (Endelman and Jannink 2012), and *σ*^2^_a_ is the estimated additive genetic variance (Yu et al. 2006). For each model, we calculated ***K*** using SNP data from the genotyped lines included in the model with the “rrBLUP” (Endelman and Jannink 2012) package in R (R Core Team 2020). For across-year GWA, the residual variance was modeled as ***Iw****σ*^*2*^_*ε*_, where ***w*** is the vector of genotype BLUE variances (Möhring and Piepho 2009; George and Cavanagh 2015; Xue et al. 2017). The variance components were estimated once for each GWA model using the “population parameters previously determined” (P3D) method (Zhang et al. 2010).

Although ***K*** was included in all GWA models to account for population structure (Yu et al. 2006), there was little evidence of population structure in the breeding panel. We conducted a principal component analysis of the 5233 genotyped lines using SNP data with the “FactoMineR” (Lê et al. 2008) package in R (R Core Team 2020). The first and second principal components accounted for 4.0% and 3.5% of the variance, respectively, and demonstrated some separation among lines with respect to the first year in which they appeared in the population (Online Resource 4).

SNP *p* values and effect estimates were extracted from each GWA model. For multiple test correction of the SNP p values, we conducted a false discovery rate (*α* = 0.05) analysis for each GWA model with the “qvalue” package (Storey 2015) in R (R Core Team 2020). The “sommer” package estimates SNP effect estimates (*β*) as $\beta = \left( {X^{\prime } V^{ - } X} \right)X^{\prime } V^{ - } y$ with $X = ZM_{i}$, where *Z* is the incidence matrix of the genotype random effect, *M*_*i*_ is the *i*th column of the SNP matrix, $V^{ - }$ is the inverse of the phenotypic variance matrix, and *y* is the response (Covarrubias-Pazaran 2016). Because SNPs were coded as − 1 = aa, 0 = Aa, and 1 = AA, *β* is always relative to the number of “A” alleles.

### Tests for selection

For each SNP, we calculated the frequency of allele “A” (*p*) in each year from 2008 to 2018 and extracted *β* from each within-year GWA model. To estimate the change in allele frequency of each SNP from 2008 to 2018, we fit a linear model for each SNP with *p* as the response and year as a continuous fixed effect and extracted the year coefficient from the model (*Δp*). Likewise, we estimated the change in allele effect of each SNP from 2008 to 2018 by fitting a linear model for each SNP with *β* as the response and year as a continuous fixed effect and extracted the year coefficient from the model (*Δβ*). In GWA, the power to detect a SNP-trait association and the absolute effect size of a SNP decrease with decreasing minor allele frequency (Bush and Moore 2012; Xiao et al. 2017). As such, effect sizes (1) become less negative (increase) as the major resistance allele increases in frequency and (2) become less positive (decrease) as the major susceptibility allele increases in frequency. To determine whether the frequency of the resistant allele or the susceptible allele of each SNP increased over time, we used the following criteria: (1) if *Δp* > 0 and *Δβ* < 0, there was selection for the “A” allele conferring susceptibility; (2) if *Δp* > 0 and *Δβ* > 0, there was selection for the “A” allele conferring resistance; (3) if *Δp* < 0 and *Δβ* < 0, there was selection for the alternate “a” allele conferring resistance; (4) if *Δp* < 0 and *Δβ* > 0, there was selection for the “a” allele conferring susceptibility.

We sought to test whether changes in allele frequencies were driven by selection rather than drift. For each SNP, we calculated the observed variance in allele frequency from 2008 to 2018 (*V*_*p*_) and estimated the expected variance in allele frequency due to random genetic drift (*V*_*t*_*)* as $V_{t} = p\left( {1 - p} \right)\left( {1 - {\text{exp}}\left( { - t/2N_{{\text{e}}} } \right)} \right)$, where *p* is the initial “A” allele frequency in 2008, *t* is the number of generations (*t* = 11 generations from 2008 to 2018), and *N*_e_ is the effective population size (Ridley 2003; Juliana et al. 2019). We estimated *N*_e_ (*N*_e_ = 149) by regressing identity-by-descent (IBD) coefficients against time (2008–2018), with $N_{{\text{e}}} = 1/2\Delta IBD$ (Falconer and Mackay 1995). For each year (2008–2018), we calculated IBD between all pairs of lines using the SNPRelate package (Zheng et al. 2012) in R (R Core Team 2020). For each SNP, we then calculated the difference between the observed and expected variances, *V*_*p *_– *V*_*t*_. We compared *V*_*p *_– *V*_*t*_ of each SNP to the genome-wide null distribution of *V*_*p *_– *V*_*t*_. The null distribution was generated by subsampling *V*_*p *_– *V*_*t*_ from 150 random SNPs in 1000 replications. The subsample size of 150 was selected because there were 150 significantly associated SNPs from GWA.

To test for genome-wide selection of stripe rust resistance or susceptibility, we estimated $\hat{G}$, a composite statistic of the relationship between additive effect estimates and allele frequency changes over time of genome-wide markers, as described by Beissinger et al. (2018). We fit a random regression best linear unbiased prediction (rrBLUP) model with genotype BLUEs as the response (as described in the two-stage across-year GWA) and SNPs as fixed effects using the “rrBLUP” package (Endelman 2011) in R (R Core Team 2020). For each SNP, we extracted its estimated effect from the rrBLUP model and *Δp* (change in allele frequency from 2008 to 2018) from the selection analysis. We then estimated the value and significance of $\hat{G}$ with 1000 permutations using the “Ghat” package (Beissinger et al. 2018) in R (R Core Team 2020). As described by Beissinger et al. (2018), $\hat{G} = \sum\nolimits_{j = 1}^{m} {\Delta_{j} } \alpha_{j}$, where *Δ*_*j*_ is the change in allele frequency from 2008 to 2018 for SNP *j*, *α*_*j*_ is the allele effect of SNP *j*, and *m* is the total number of SNPs. To test whether the observed $\hat{G}$ was the result of selection rather than drift, $\hat{G}$ was compared to the null distribution of $\hat{G}_{{{\text{perm}}}}$ (Beissinger et al. 2018). SNP allele effects were permuted 1000 times, and $\hat{G}_{{{\text{perm}}}}$ was estimated for each permutation as $\hat{G}_{{{\text{perm}}}} = \sum\nolimits_{j = 1}^{m} {\Delta_{j} } \alpha_{{p_{j} }}$, where *Δ*_*j*_ is the change in allele frequency from 2008 to 2018 for SNP *j*, $\alpha_{{p_{j} }}$ is the allele effect of permuted SNP *j*, and *m* is the total number of SNPs (Beissinger et al. 2018). In this study, a negative $\hat{G}$ indicates selection for resistance to stripe rust and a positive $\hat{G}$ indicates selection for susceptibility.

## Results

### Genotypic and trial effects on and heritability for stripe rust

From 2008 to 2012, stripe rust was evaluated on 962–1789 genotypes in one trial per year (Table 1). Stripe rust was evaluated on a larger panel of genotypes (1465–4134) in a greater number of trials (2–24) per year from 2013 to 2018 (Table 1). Broad-sense heritability (*H*^2^) for resistance to stripe rust was generally high within years (*H*^2^ = 0.50–0.90) and was moderate across years (*H*^2^ = 0.54) (Table 2). In most years, genotype explained a larger amount of the variance in stripe rust than trial and/or error (Table 2).

**Table 2:** Number (*N*) of plots, genotypes, and trials, variance components, and broad-sense heritability (*H*^2^) from phenotypic analysis of stripe rust resistance within and across years from 2008 to 2018

Year	Plots	Genotype		Trial^a^		Error	*H*^2^
	*N*	*σ*^2^_g_	*N*	*σ*^2^_t_	*N*	*σ*^2^_ε_	
2008	1103	1.424	962			0.332	0.86
2009	2065	1.836	1177			0.606	0.84
2010	2325	1.699	1672			0.469	0.83
2011	2915	0.322	1789			0.387	0.56
2012	3072	1.067	1701			0.308	0.85
2013	3421	0.397	1468	0.376	3	0.062	0.90
2014	12,363	2.106	3353	0.784	24	1.020	0.78
2015	11,485	1.701	4134	0.446	17	0.717	0.76
2016	11,188	1.227	3848	2.377	14	1.830	0.50
2017	4037	1.041	1465	1.124	6	0.812	0.68
2018	5425	0.375	3507	0.001	2	0.049	0.90
Across	59,399	1.010	20,529	0.845	71	1.165	0.54

^a^The trial term was not included in within-year models for 2008–2012 because there was only one trial per year

Between years, genotype BLUPs for stripe rust were positively correlated (Table 3). The number of genotypes in common was larger and phenotypic correlations tended to be stronger in adjacent years than in more distant years (Table 3). The highest correlations were observed between pairs of years from 2008 to 2012 (Table 3), where stripe rust was evaluated under artificial inoculation in the disease nursery in Reichersberg, Austria. From 2013 to 2018, trials were both artificially inoculated and naturally infected and were conducted in several locations.

**Table 3:** Correlations between genotype best linear unbiased predictors for stripe rust from 2008 to 2018

*n*\*r*	2008	2009	2010	2011	2012	2013	2014	2015	2016	2017	2018
2008	962	0.77***	0.81***	0.65***	0.68**	0.36	0.52**	0.25	0.61*	0.62*	0.85**
2009	188	1177	0.76***	0.53***	0.59***	0.40*	0.21	0.49**	0.58**	0.51*	0.46
2010	76	421	1672	0.47***	0.68***	0.29*	0.26	0.26	0.34	− 0.02	0.50*
2011	61	98	225	1789	0.38***	0.42***	0.02	− 0.05	0.25	0.04	0.47**
2012	43	56	84	496	1701	0.45***	0.17	0.27*	0.15	0.31*	0.24
2013	24	38	49	149	260	1468	0.27**	0.49***	0.19	0.47**	0.16
2014	34	41	48	81	111	171	3353	0.54***	0.13	0.20*	0.24
2015	28	33	40	62	72	80	690	4134	0.13**	0.25***	0.30**
2016	14	22	30	41	55	58	219	734	3848	0.20***	0.36***
2017	14	19	27	36	46	44	152	261	925	1465	0.53***
2018	12	18	23	34	38	40	64	110	190	217	3507

Correlation coefficients (*r*) and *p* values are in the upper diagonal. Number of genotypes (*n*) present in each pair of years is in the lower diagonal. Number of genotypes within each year is on the diagonal. *p* values are denoted as *0.05 < *p* ≤ 0.01; **0.01 < *p* ≤ 0.0001; *p* < 0.0001

### Genome-wide association of stripe rust resistance

**Fig. 1:**
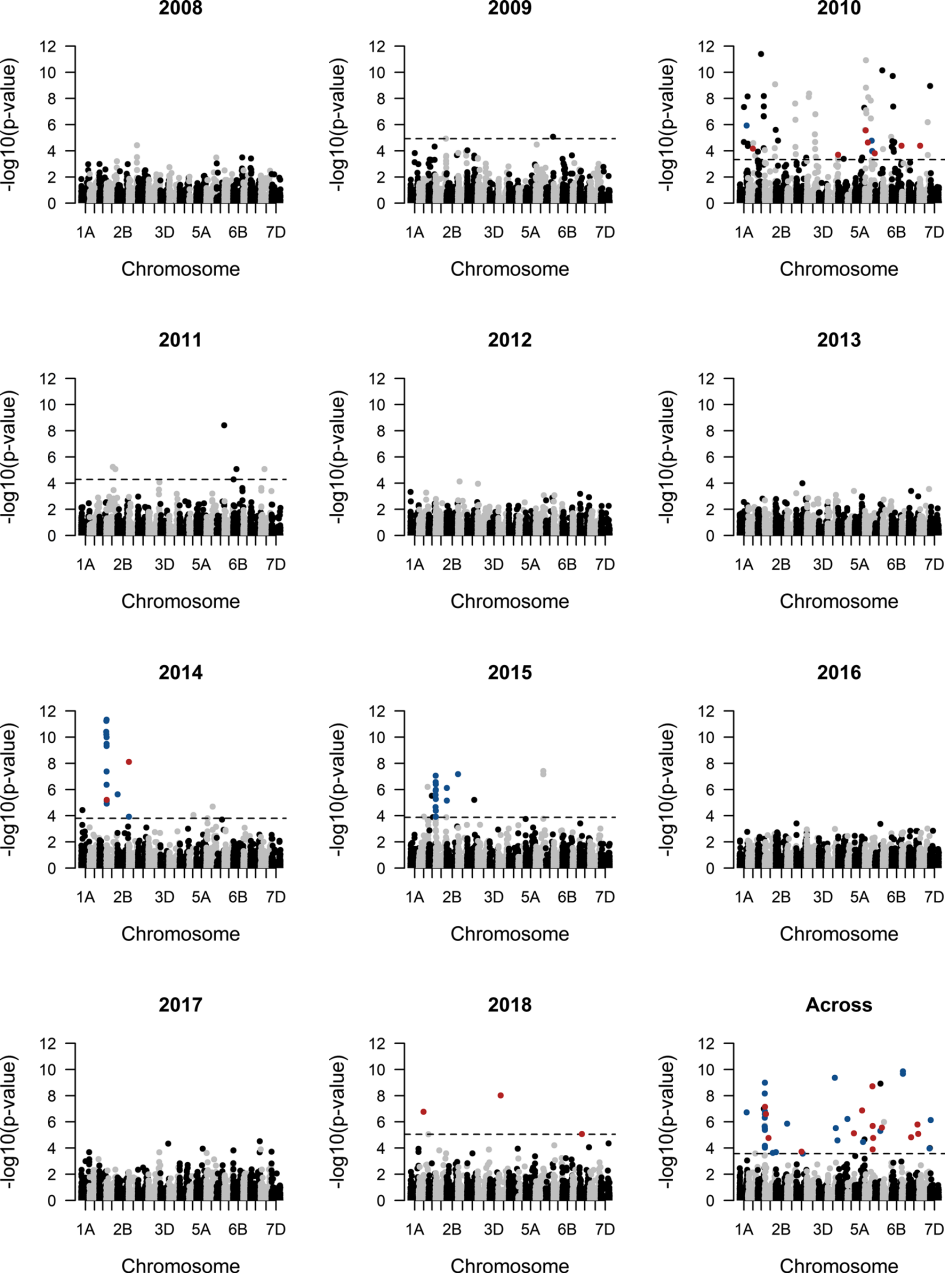
Manhattan plots of GWA for stripe rust within (2009–2018) and across (2008–2018) years, with SNP physical positions on the *x*-axis, SNP − log_10_(*p* values) on the y-axis, and dashed horizontal lines denoting the FDR threshold for SNP significance. SNPs highlighted in blue and red denote SNPs under selection for the resistant and susceptible allele, respectively

GWA across years and within 2009–2011, 2014–2015, and 2018 revealed 186 significant SNP-stripe rust associations (150 unique SNPs) after multiple test correction (Fig. 1, Online Resource 5–6). Of the significantly associated SNPs, 112 had a positive effect (“A” allele confers susceptibility) and 38 had a negative effect (“A” allele confers resistance) on stripe rust (Online Resource 6). The significant GWA SNPs explained a small proportion of the variance in stripe rust (*R*^2^ = 0.08 ± 0.12) and had small to medium-effect sizes (|*β*|= 1.09 ± 1.23) (Online Resource 6).

QTL colocalized between models at 12 locations (Fig. 1, Online Resource 6). The within-2010 and across-year GWA shared a SNP on chromosome 1A at 499.7 Mbp (Fig. 1, Online Resource 6). One SNP on chromosome 1D at 234.1 Mbp was significant in GWA within 2010 and 2015 (Fig. 1, Online Resource 6). The 2014 and 2015 analysis shared a SNP on chromosome 2A at 1.8 Mbp (Fig. 1, Online Resource 6). SNPs from GWA across years and within 2010, 2014, and 2015 colocalized on chromosome 2A at 3.4–16.5 Mbp (Fig. 1, Online Resource 6). GWA across years and within 2014 and 2015 shared SNPs on chromosome 2A at 18.8–21 Mbp (Fig. 1, Online Resource 6). The across-year and within-2014 analysis shared a SNP chromosome 2A at 31.4 Mbp (Fig. 1, Online Resource 6). A SNP on chromosome 2A at 739.3 Mbp was found in GWA in 2009 and 2010 (Fig. 1, Online Resource 6). GWA across years and in 2014 and 2015 had colocalized SNPs on chromosome 2B at 23.1 and 24.8 Mbp and on chromosome 2D at 4.3 Mbp (Fig. 1, Online Resource 6). Across-year and within-2010 GWA shared a SNP on chromosome 5A at 522.5 Mbp (Fig. 1, Online Resource 6). A SNP on chromosome 5D at 528.7 Mbp was significantly associated in 2010 and 2014 (Fig. 1, Online Resource 6). A SNP on chromosome 7A at 176.8 Mbp was significantly associated in both the 2018 and across-year GWA (Fig. 1, Online Resource 6).

No SNPs were significantly associated with stripe rust in 2008, 2012–2013, and 2016–2017 (Fig. 1, Online Resource 5). Quantile–quantile plots of the expected versus the observed *p* values from each GWA demonstrated that the analysis was underpowered in years in which no SNPs were identified (Online Resource 7). Few genotypes (*N* = 47) had SNP data in 2008 and the trial and residual terms explained a larger proportion of the variance in stripe rust in years with no significantly associated GWA SNPs, which may partially explain the lack of statistical power to detect SNP-trait associations (Online Resource 5).

### Evidence of selection for stripe rust resistance

**Fig. 2:**
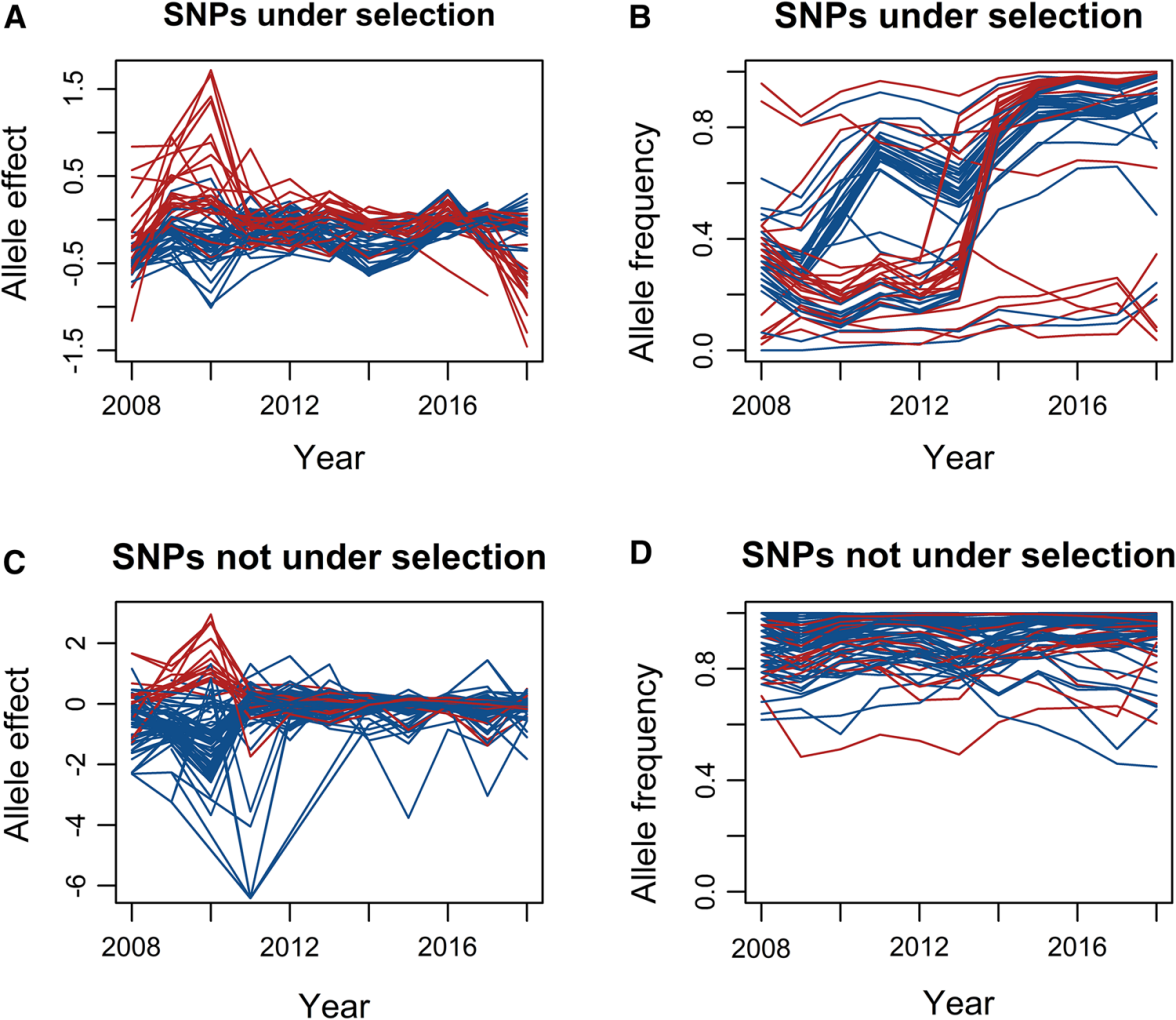
Allele effects and allele frequencies of SNPs significantly associated in GWA for stripe rust from 2008 to 2018. For SNPs with significant evidence of selection, the effect (**A**) and frequency (**B**) of the allele under selection (regardless of “A” or “a” allele state) are plotted against time, with SNPs with selection for the resistant allele in blue and for the susceptible allele in red. For SNPs not under selection, the effect (**C**) and frequency (**D**) of the major allele (allele at higher frequency, regardless of “A” or “a” allele state) are plotted against time, with blue and red denoting resistance and susceptibility conferred by the major allele, respectively

We assessed changes in allele frequencies and allele effects on stripe rust for each SNP from 2008 to 2018 and tested whether these changes were driven by selection or random genetic drift (Online Resource 8). By comparing the variance in observed allele frequencies to the expected variance due to drift (|*V*_*p *_– *V*_*t*_|) of each SNP against the null distribution of |*V*_*p *_– *V*_*t*_| (bootstrapped 1000 times, 95% quantile = 0.0008), we found significant evidence of selection of the resistant allele at 38/150 significant GWA SNPs (“A” allele at 23 SNPs; “a” allele at 15 SNPs) and for selection of the susceptible allele at 24/150 significant GWA SNPs (“A” allele at 8 SNPs; “a” allele at 16 SNPs) (Figs. 1, 2, Online Resource 8).

SNPs significantly associated in GWA and in selection tests demonstrated sharp changes in allele frequencies from 2012 to 2013, suggesting increased selection pressure during the generation between 2012 and 2013; the resistant or susceptible allele of these SNPs was nearly fixed in the population by 2018 (Fig. 2, Online Resource 8). In contrast, the allele frequencies and effects of the significant GWA SNPs that were not under selection were relatively unchanged from 2008 to 2018. Of the 88 significant GWA SNPs not under selection, the major allele (allele at higher frequency, regardless of “A” or “a” allele state) conferred resistance at 71 SNPs and susceptibility at 17 SNPs (Fig. 2, Online Resource 8). Allele effect estimates may have been inflated in 2008–2012, as stripe rust was only evaluated in one trial per year and fewer genotypes had SNP data in these years than in 2013–2018 (Fig. 2).

**Fig. 3:**
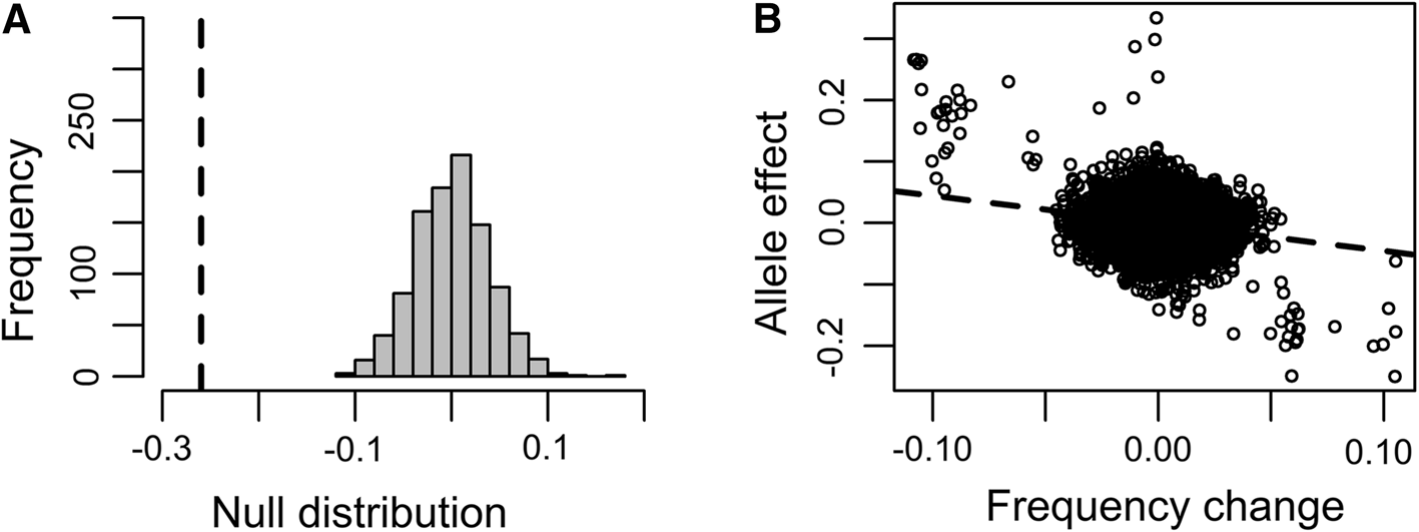
(**A**) Histogram of the null distribution of 1000 permuted Ghat values ($\hat{G}_{{{\text{perm}}}}$) and the observed Ghat value ($\hat{G})$ plotted as a dashed vertical line and (**B**) plot of allele effects on stripe rust from across-year GWA versus allele frequency changes from 2008 to 2018

To test whether genome-wide resistance or susceptibility to stripe rust was under selection in the breeding program between 2008 and 2018, we used the $\hat{G}$ method (Beissinger et al. 2018). There was significant evidence of genome-wide selection for stripe rust resistance over the 11 years in the population, as demonstrated by a negative $\hat{G}$ value ($\hat{G}$ = − 0.26) and a highly significant (*p* = 2 × 10^–16^) difference between the observed $\hat{G}$ and the null distribution of 1000 permuted $\hat{G}_{{{\text{perm}}}}$ values (Fig. 3A).

SNPs with larger effect sizes on stripe rust in across-year GWA had greater changes in allele frequencies from 2008 to 2018 (Fig. 3B). Furthermore, 2483 SNPs were not significantly associated with stripe rust in GWA within or across years, yet they had significant evidence of selection for the resistant (1233 SNPs) or the susceptible (1250 SNPs) allele over time (Online Resource 8). For SNPs under selection, absolute allele effect sizes from GWA within and across years (|*β*|) and *V*_*p *_– *V*_*t*_ were greater at significant GWA SNPs (|*β*|= 0.42 ± 0.36; *V*_*p *_– *V*_*t*_ = 0.07 ± 0.06) than at nonsignificant GWA SNPs (|*β*|= 0.12 ± 0.15; *V*_*p *_– *V*_*t*_ = 0.005 ± 0.006). These results suggest that both moderate and small effect QTL were under selection, although to a lesser extent for QTL with small effects that were not detectable GWA.

## Discussion

Selection pressure for stripe rust resistance can be influenced by both breeder’s decisions and changes in pathotype composition of *Pst* populations. Here, we combined quantitative genetic and population genetic methods to identify genomic regions that were under selection for resistance or susceptibility to stripe rust in an Austrian winter wheat breeding program from 2008 to 2018. GWA revealed 150 SNPs significantly associated with stripe rust within 2009–2011, 2014–2015, and 2018 and across 2008–2018, many of which overlapped with regions previously associated with stripe rust resistance in other populations (Rosewarne et al. 2013) and with putative *Yr* R-genes (Waqar et al. 2018; Blake et al. 2019). Because the ability to detect SNP-trait associations is largely dependent on minor allele frequency (Bush and Moore 2012; Xiao et al. 2017) and selection within a breeding program can generate rapid changes in allele frequencies (Ridley 2003), the majority of these SNPs were detected by GWA in only 1 year or in adjacent years. Investigating the dynamics in allele frequencies and effects over time can identify regions of the genome which have undergone selection for specific traits (Juliana et al. 2019). By combining GWA and selection testing, we found that both small- and moderate-effect loci had evidence of selection in the population. We also employed the $\hat{G}$ method to assess selection at the genome-wide level (Beissinger et al. 2018) and found that the breeding population demonstrated genome-wide selection for resistance from 2008 to 2018.

The highly significant QTL on the short arm of chromosome 2A were under selection for resistance and although it was physically near the *Yr17* gene (Rosewarne et al. 2013), it is unlikely that *Yr17* underlies this QTL because virulent *Pst* races overcame *Yr17* across European wheat cultivars by 2000 (Bayles et al. 2000). Two significant GWA SNPs on the short arm of chromosome 2B also demonstrated selection for the resistant allele and may be linked to *Yr27*, an R-gene which has recently broken down against new *Warrior*-type races of *Pst* in the Middle East (Tehseen et al. 2020). A SNP in the pericentromeric region of chromosome 1A was under selection for the resistant allele and was near QTL for adult plant resistance to stripe rust from four mapping populations (Rosewarne et al. 2008; Dedryver et al. 2009; Bariana et al. 2010; Ren et al. 2012) and in a panel of elite spring wheat lines from CIMMYT (Crossa et al. 2007), but no *Yr* genes have been mapped to this region (Waqar et al. 2018; Blake et al. 2019). Four SNPs on the long arm of chromosome 5B were under selection for susceptibility and colocalized with QTL associated with non-race-specific adult plant resistance to stripe rust in a Sichuan wheat diversity panel (Ye et al. 2019) and with QTL for race-specific seedling resistance to stripe rust found in two biparental mapping populations (Feng et al. 2011; Zegeye et al. 2014) and for adult plant resistance in an Austrian biparental mapping population (Buerstmayr et al. 2014). However, no *Yr* genes have been mapped to the long arm of chromosome 5B to date (Waqar et al. 2018; Blake et al. 2019). Two SNPs on the long arm of chromosome 7A were also under selection for susceptibility, but we found no evidence of previously reported stripe rust QTL or mapped *Yr* genes in this region (Waqar et al. 2018; Blake et al. 2019).

By combining SNP-specific and genome-wide approaches, we demonstrated that the breeding population harbors both moderate-effect QTL and quantitative forms of incomplete, race non-specific adult plant resistance and that both were under selection across the 11-year period. The resistance QTL identified in this study will be further evaluated for their use in marker-assisted selection and as covariates in genomic prediction models for stripe rust resistance in the breeding program. The breeding population demonstrated highly heritable, quantitative resistance to stripe rust and low population structure, indicating that genomic prediction of stripe rust resistance can be successfully applied in this population (Crossa et al. 2017; Juliana et al. 2017; Muleta et al. 2017; Tehseen et al. 2021).

## Supplementary Information

Below is the link to the electronic supplementary material.

**Online Resource 1**. Data for each plot, including trial ID, trial year, trial location ID, row and column positions, genotype ID, raw and spatially-adjusted stripe rust phenotypes, and timepoint of stripe rust scoring (CSV 2553 kb)

**Online Resource 2**. Data for 9744 SNPs, including SNP ID, chromosome, physical position (bp), and genotypes of 5233 lines (CSV 116263 kb)

**Online Resource 3**. Genotype best-linear unbiased estimates (BLUEs) and variances used in two-stage across-year GWA (CSV 655 kb)

**Online Resource 4**. Plot of first and second principal components from principal component analysis of the breeding panel using SNP data. Each point represents one breeding line and is colored based on the year in which the line first appeared in the panel (TIF 34804 kb)

**Online Resource 5**. Number of observations, variance and number of genotypes, variance and number of trials, error variance, false discovery rate (FDR, α = 0.05) SNP p-value significance thresholds (NA = no significant SNPs), and number of significant SNPs from genome wide association of stripe rust resistance within and across years from 2008 to 2018 (CSV 0 kb)

**Online Resource 6**. GWA results for each SNP tested within each year or across years from 2008-2018, including chromosome, physical position (Mbp), effect estimate, p-value, F-statistic, and R^2^ (CSV 6525 kb)

**Online Resource 7**. Quantile-quantile plots of the expected vs. the observed SNP p-values from GWA for stripe rust within and across years from 2008 to 2018 (TIF 266227 kb)

**Online Resource 8**. Selection tests on 9744 SNPs, including allele frequencies and effects from GWA within 2008–2018, changes in allele frequencies and effects from 2008 to 2018, observed variance in allele frequencies from 2008 to 2018, expected variance in allele frequencies from 2008 to 2018 due to drift, difference between observed allele frequency variance and expected variance due to drift, selected effect (resistant/susceptible), selected allele (A/a), and significance in GWA (CSV 3119 kb)

## Data Availability

All phenotypic and genotypic data and results from the analyses presented here are included in the manuscript materials.

## Code availability

The scripts used to conduct the analyses presented here are available upon request.
