# Supplementary figures and images for: Genomic signatures of selection for resistance to stripe rust in Austrian winter wheat
Source: Theor Appl Genet. 2021 Jun 14;134(9):3111–21. doi: 10.1007/s00122-021-03882-3 (PMC8354948; doi:10.1007/s00122-021-03882-3)

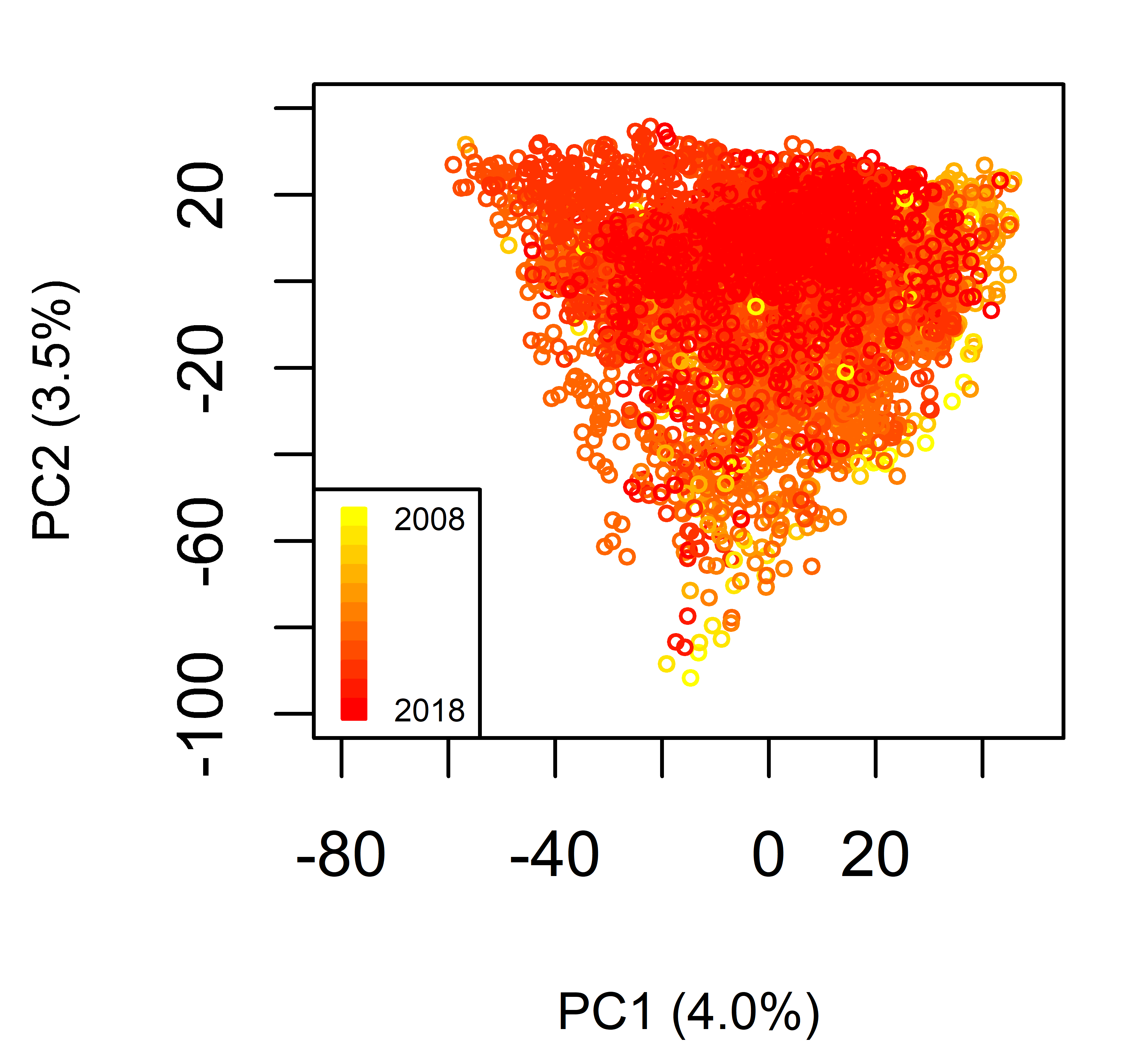

Supplement: Supplementary file 4 — Online Resource 4. Plot of first and second principal components from principal component analysis of the breeding panel using SNP data. Each point represents one breeding line and is colored based on the year in which the line first appeared in the panel (TIF 34804 kb) [file 122_2021_3882_MOESM4_ESM.tif]
